# Assessment of seawater bacterial infection in rabbit tibia by Illumina MiSeq sequencing and bacterial culture

**DOI:** 10.1186/s13018-021-02553-9

**Published:** 2021-07-21

**Authors:** Du Wang, Qingcong Zheng, Qi Lv, Chaofan Zhang, Yun Zheng, Huidong Chen, Wenming Zhang

**Affiliations:** 1 Department of Joint Surgery, The First Affiliated Hospital of Fujian Medical University, Fuzhou, China; 2 Department of Orthopedics, 900th Hospital of Joint Logistics Support Force, Fuzhou, China

**Keywords:** Scanning electron microscopy, Sequencing, Marine, New Zealand rabbit, Bacterial infection

## Abstract

**Objectives:**

We aimed to explore the bacterial community composition following ocean bacterial infection using an animal model.

**Methods:**

This animal-based experiment was conducted from September 2019 to November 2019. Eighteen seawater filter membranes were collected from Changle City, Fujiian Province, China, on September 8, 2019. Ten filter membranes were used for implantation. Eight filter membranes that were used in the bacterial culture for the exploration of seawater bacteria were assigned to the seawater group (SG). Fourteen healthy adult New Zealand rabbits were randomly divided into the experimental group (EG) and control group (CG). Seawater filter membranes and asepsis membranes were implanted into the tibia in the EG and CG, respectively. One week after surgery, tibial bone pathology tissues were collected and assessed using light microscopy and scanning electron microscopy (SEM). Medullary cavity tissues were collected for the performance of Illumina MiSeq sequencing and bacterial culture. The differences between EG and CG were assessed by pathological observation under light microscopy and SEM, high-throughput bacterial sequencing, and bacterial culture.

**Results:**

Compared with the CG, the infection rate was 100%, and the mortality value was 20% after the implantation of the filter membranes in the EG. Both light microscopy and SEM showed that a large number of bacteria were distributed in the bone marrow cavity after ocean bacterial infection. No bacterial growth was found in the CG. Illumina MiSeq sequencing found that *Firmicutes*, *Proteobacteria*, *Thermotogae*, *Fusobacteria*, *Bacteroidetes*, and *Actinobacteria* were the dominant bacteria at the phylum level and *Clostridium*_sensu_stricto_7, *Haloimpatiens*, *Clostridium*_sensu_stricto_15, *Clostridiaceae*_1, *Clostridium*_sensu_stricto_18, and *Oceanotoga* were the dominant bacteria in genus level among the EG. In the bacterial culture of the medullary cavity tissues, *Klebsiella pneumoniae*, *Shewanella algae*, *Staphylococcus aureus*, *Escherichia coli*, *Enterobacter cloacae,* and *Vibrio vulnificus* were the predominant infective species. Moreover, compared with the SG, the EG showed a higher detection rate of *E. coli* and *S. aureus* (*P* = 0.008 and *P* = 0.001, respectively). The detection rates of *V. alginolyticus*, *V. parahaemolyticus*, and *V. fluvialis* were higher in the SG than the EG (*P* = 0.007, *P* = 0.03, and *P* = 0.03, respectively).

**Conclusions:**

Our model, which was comprehensively evaluated using four techniques: histopathology and SEM observation, gene detection, and bacteria culture, provides a scientific basis for the clinical diagnosis and treatment of patients in such settings.

**Supplementary Information:**

The online version contains supplementary material available at 10.1186/s13018-021-02553-9.

## Introduction

In recent years, ocean tourism has gained momentum, with nearly 40% of all tourists, globally, showing a tendency to visit coastlines along the Atlantic Ocean in Western Europe and North America [1, 2]. With the increase in the levels of contact with ocean water, the rates of wound infections due to recreational activities near these water sources were also higher than average [3]. In addition, the development of the fishery industry and marine resource exploitation have led to an increase in the risk of seawater bacterial infection.

Bacterial infection is among the most commonly observed complications in the field of orthopedics [4]. The degree of bacterial infection is closely related to the injury mechanism, injury degree, host, and post-traumatic treatment [5, 6]. However, the species, degree, and characteristics of the bacteria observed after seawater immersion in patients with limb injury are significantly different from those noted in patients on land. Moreover, the physical properties of seawater, such as its hypertonic nature, high sodium level, alkalinity, and low temperature, directly affect the species composition. The most commonly observed bacterial infections following an open fracture on land are caused by *Pseudomonas aeruginosa*, *Acinetobacter baumannii, Enterobacter*, and methicillin-resistant *Staphylococcus aureus,* none of which exist in wounds soaked in seawater [7]. These differences may be attributed to two factors: first, bacterial infections may be associated with differences in the causative species and their quantity, virulence, and invasiveness; second, people living on land for a long time have different body reactions to marine bacteria, in terms of the time, degree, and scope of infection, among others. Before 1980, wound bacterial culture was routinely performed before surgical debridement [8, 9]. However, recent studies have shown that since the pathogens observed in general wound infections on land are usually acquired in the hospital, wound bacterial culture before debridement for the prediction of wound infection after debridement is not as valuable [10, 11]; therefore, cephalosporins are often used in the early stage [12, 13]. While the correlation between pathogenicity and seawater bacteria is strong after open trauma following seawater immersion, the correlation with pathogens in the hospital is weaker [7]. The detection of seawater bacteria is important for the prediction of the pathogens that may present postoperatively.

However, few studies have explored the bacterial community composition of bone infection caused by seawater bacteria. Therefore, this study aimed to (a) explore infection and mortality rate of marine bacteria in rabbit tibial model and observe infectious tissue by optical microscope and SEM, (b) assess bacterial community composition of infective rabbit tibia by Illumina MiSeq sequencing, and (c) culture marine pathogenic bacteria from infected rabbit tibia and seawater.

## Materials and methods

### Seawater collection

Seawater samples were collected from the coastal region of Xiangbi Bay, Changle City, Fujian Province, China (25.9633° N, 119.7104° E) on September 8, 2019. The suction filtration system was used to filter 40 × 500 mL seawater samples with filter membranes (pore diameter 0.22 μm, diameter 50 mm, mixed cellulose esters, China), and 18 seawater filter membranes with seawater bacteria were finally obtained. Ten filter membranes were used for surgery implantation, and the remaining eight, which were used in the bacterial culture for the investigation of seawater bacteria, were assigned to the seawater group (SG).

### Experimental animals

Totally, 14 healthy adult New Zealand rabbits were provided by the Experimental Animal Center of the 900th Hospital of the Joint Logistics Support Force; they had a body mass of 2.5 ± 0.2 kg. Fourteen New Zealand rabbits were randomly numbered from 1 to 14 and randomly divided into two groups by a computer: an experimental group (EG) (*n* = 10) (EG13 died during anesthesia) and a control group (CG) (*n* = 3).

### Operative procedure

Surgery was performed under general anesthesia with 1% pentobarbital sodium (30 mg/kg body weight). The left hind limb of each animal was shaved and marked, and the limbs of each animal were fixed on the operating table in a supine position. After skin disinfection, an incision, 2 cm in size, was longitudinally made between the tibial tubercle and medial collateral ligament. The skin, subcutaneous tissue, and fascia were cut open, and the muscles were separated to expose the medial bone surface of the tibia. An electric drill with a diameter of 8 mm was used to create a hole on the bone surface to access the medullary cavity at the distal tibia. In the EG, the seawater filter membrane was filled into the medullary cavity. The drill hole was sealed with bone wax, and the incision was sutured completely and bandaged with pressure. In the CG, the asepsis filter membrane was placed in the medullary cavity in the same manner.

Following the operation, the wounds of each animal were treated with sterile gauze and bandage, which were changed every day; however, no antibiotic treatment was provided. The rabbits’ vital signs and wound conditions, including the presence of swelling, pus, and ulcerations in each group, were observed every day. The infection and mortality values were evaluated. The animals that died within 1 week after the operation were observed and sampled at the time of death.

### Evaluation of tibial bone pathology

One week after the surgery, the animals in each group were sacrificed, and their tibias were completely removed. A rongeur was used to cut the tibia from the middle portion. The two parts of the tibia were cut symmetrically and placed into an aseptic culture cup, following which the specimens underwent histopathological examination and scanning electron microscopy (SEM). The tissues and filter membranes in the medullary cavity were removed and placed in an aseptic tube. The medullary cavity tissues obtained from each animal in both groups were subjected to high-throughput sequencing and bacterial culture.

### Observation indicators

#### Light microscopy observation

All bone specimens were fixed in 4% paraformaldehyde for 72 h, decalcified in 5% formic acid decalcification solution for 12 h, and embedded in paraffin, following which 4-mm longitudinal sections were cut using a microtome. The sections were stained with hematoxylin & eosin (HE) and observed under a light microscope.

#### SEM observation

The bone samples obtained from each group were fixed in a sterile culture cup containing 2.5% glutaraldehyde for 2 h; washed with 0.1 mol/L phosphate-buffered saline thrice for 15 min each time; then dehydrated with 30%, 50%, 70%, 80%, and 90% ethanol for 10 min each; and finally dehydrated with 100% anhydrous ethanol thrice, for 10 min each time. The samples were then placed in a critical point dryer (K850, Emitech, UN) and then in a Sputter Coater (Q150R, Quorum, UN). Finally, the tibia specimens were observed by SEM (Quanta 450 FEG, Thermo Fisher Scientific, USA).

#### Illumina MiSeq sequencing

DNA extraction was conducted using the E.Z.N.A.® Soil DNA kit (Omega Bio-tek, Norcross, GA, USA) following the manufacturer’s protocols. Afterwards, the quality of the DNA samples was assessed using 1.0% agarose gel electrophoresis, and the concentration was measured with a NanoDrop spectrophotometer. Hypervariable regions V3–V4 of bacterial 16S rRNA genes were amplified using barcode and adaptor added primer 338F (5′-ACTCCTACGGGAGGCAGCAG-3′) and 806R (5′-GGACTACHVGGGTWTCTAAT-3′) [14, 15]. Polymerase chain reaction (PCR) was performed in triplicate in 20-μl reaction volumes, containing 4 μl 5*FastPfu buffer, 2 μl 2.5 mM dNTPs, 0.8 μl primer (5 μM), 0.4 μl FastPfu polymerase, and 10-ng DNA template. The reaction condition was set at an initial denaturation level of 95 °C for 3 min, followed by 27 cycles of 95 °C for 30 s, 55 °C for 30 s, and 72 °C for 30 s. The final extension was performed at 72 °C for 10 min.

We used 2% agarose gel to recover the PCR products, which were then purified using an AxyPrep DNA Gel Extraction Kit (AxygenBiosciences, UnionCity, CA, USA), with Tris-HCl elution, and quantified with QuantiFluor™-ST (Promega, USA). According to the standard operating procedures of the Illumina MiSeq platform (Illumina, San Diego, USA), the purified amplified fragments were pooled in equimolar ratios and subjected to paired-end sequencing (2 × 300) [16, 17].

The UPARSE software performs operational taxonomic unit (OTU) clustering of sequences based on a 97% similarity cut-off and removes single sequences and chimeras during the clustering process [18]. Alpha diversity estimates were calculated using mothur (v1.30.2, https://mothur.org/) with multiple indices, including Good’s coverage, alpha diversity indices (Shannon–Wiener and Simpson), richness estimators (Chao1), and abundance-based richness estimation (ACE). The RDP Classifier was employed for the analysis, and comparisons were performed against the Silva database (SSU132) using a matching threshold of 70% [19, 20].

#### Bacterial culture

Ten samples from the EG, three from the CG, and eight seawater filter membranes were cultured. The samples were added to 25 ml of alkaline peptone water by an inoc—lation loop for enrichment and cultured at 35 °C for 6–8 h. Following this, the surface culture was inoculated with thiosulfate citrate bile salt sucrose (TCBS) agar and 3% NaCl tryptic soy agar at 35 °C for 18–24 h. The samples were added to 25 ml of Rappaport-Vassiliadis Soya Broth by an inoculation loop for enrichment and cultured at 35 °C for 18–24 h. Next, the surface culture was inoculated with Salmonella–Shigella (SS) agar and China blue agar at 35 °C for 18–24 h. The samples were added to 25 ml of nutrient broth by an inoculation loop for enrichment and cultured at 35 °C for 12–18 h. Following this, the surface culture was inoculated into blood agar, chocolate agar, and MacConkey agar at 35 °C for 18–24 h. The samples were added to 19 ml of Giolitti and Cantoni Broth by an inoculation loop for enrichment, sealed with paraffin, and cultured at 35 °C for 48 h. The surface culture was then inoculated into Baird-Parker agar for 24–48 h.

The bacteria on the blood agar plate, McConkey agar plate, chocolate plate, and China blue plate were identified as *Vibrio*, *Enterobacter*, non-fermentative bacteria, *Staphylococcus*, and Gram-positive bacilli; the 3% NaCl tryptic soy agar, TCBS, SS plate, and Baird-Parker plate had *Vibrio*, *Enterobacter*, and *Staphylococcus* respectively. All the bacteria on the plates were counted after identification.

### Statistical analysis

Continuous variables are presented as means ± standard deviations (SDs), and homogeneous continuous variables using with a normal distribution were compared using the Student t test; otherwise, they were compared using the Wilcoxon rank-sum test. Categorical variables are presented as percentages and were compared using the chi-squared test. Statistical analyses were performed in R environment (http://www.r-project.org), and statistical significance was set at *P* < 0.05.

## Results

### Characteristics of the animals

Two rabbits died within 7 days in the EG. EG3 died 4 days after the operation and EG9 died 6 days after. The infection and mortality values were 100% and 20%, respectively, in the EG. The corresponding rates were both 0% in the CG.

### Tibial bone pathology

**Fig. 1:**
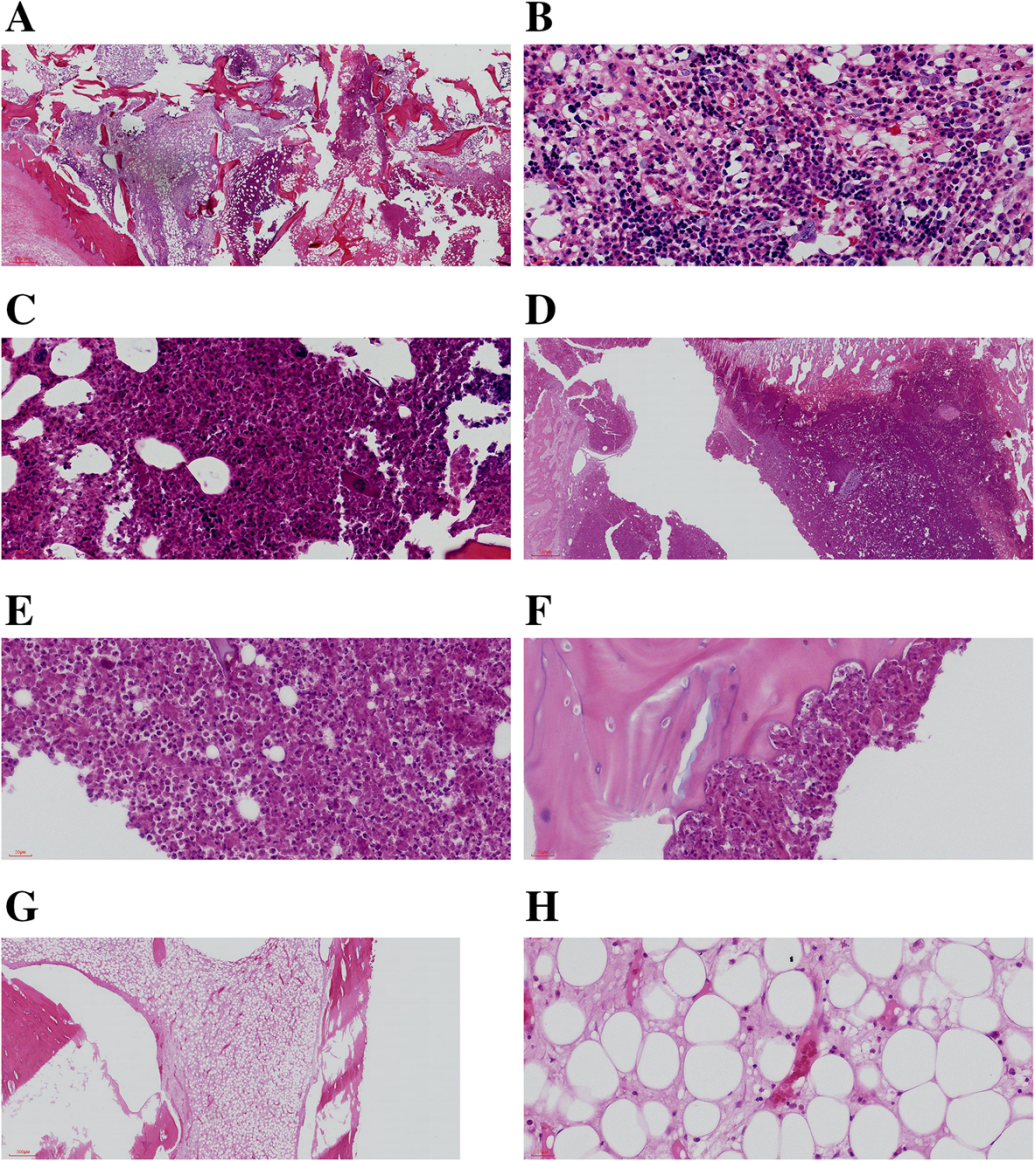
Tibial bone tissue stained with HE observed under microscope. *HE* hematoxylin and eosin. **A**, **D**, and **F** present samples from the EG, and **G** shows samples from the CG. Figures B and C are enlargements of b and c in **A**; E is an enlargement of e in **D**; **H** is the enlarged image of h in **G**. **A**, **B**, and **C** strong neutrophil, plasma cell, lymphocyte, multinucleated giant cell, and pus cell infiltration in the tibial bone marrow cavity; **D** and **E** formation of bone abscess in the metaphysis and formation of a large number of neutrophils, plasma cells, lymphocytes, multinuclear giant cells, and abscess focus in the EG. **F** inflammatory infiltration, bone erosion and destruction in the EG; **G** and **H** the hemopoietic cells and adipocytes in the bone marrow cavity are arranged regularly, and there is no obvious sign of infection in the CG (**A**, **D**, and **G**: × 20; **B**, **C**, **E**, **F**, and **H**: × 400). *EG*, experimental group; *CG*, control group

Under the light microscope, in the EG, a large number of neutrophils, plasma cells, lymphocytes, multinucleated giant cells, and purulent cells were observed in the medullary cavity (Fig. 1A, B, and C); in the CG, the hemopoietic cells and adipocytes in the medullary cavity were arranged regularly, and no obvious sign of infection was noted (Fig. 1G and H).

**Fig. 2:**
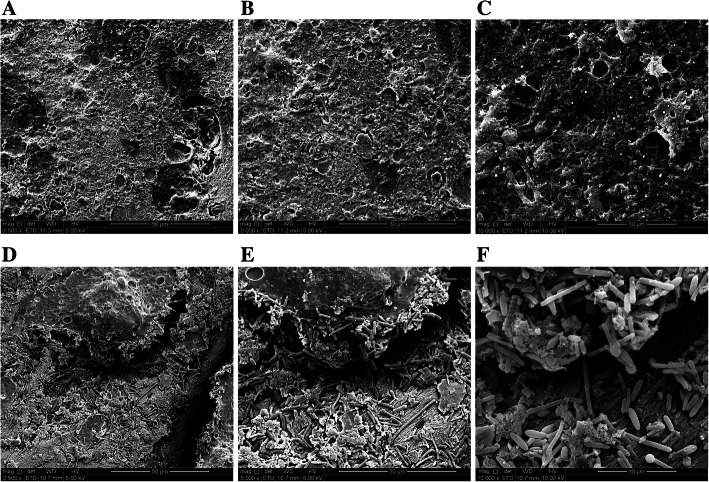
Tibial bone tissue observed by SEM in the CG and EG. **A**, **B**, and **C** The surface of the bone marrow cavity was uneven, and some cracks were found after alcohol dehydration and drying instrument treatment, but no microbial growth was observed (**A** × 2500; **B** × 5000; **C** × 10,000). **D**, **E**, and **F**: A large number of bacteria were found on the surface of the medullary cavity in a dense or scattered manner with different sizes and shapes. A large number of secretions were attached and wrapped between the microorganisms in the EG (**D** × 2500; *E* × 5000; **F** × 10,000). SEM, scanning electron microscopy; CG, control group; EG, experimental group

On SEM, no microbial growth was observed in the CG (Fig. 2A, B, and C; 2500×, 5000×, and 10,000×, respectively). However, a large number of bacteria were found on the surface of the medullary cavity, in a dense or scattered manner, and of different sizes and shapes. A large number of secretions were attached and wrapped between the microorganisms in the EG (Fig. 2D, E, and F; 2500×, 5000×, and 10,000×, respectively).

### Analysis of OTU diversity and richness

In the EG, 577893 v3–v4 sequences were obtained by MiSeq sequencing of 16S rRNA, ranging from 45,520 to 61,226. In the CG, the corresponding sequence was 105,128, ranging from 30,885 to 39,502. After random resampling with a 3% dissimilarity threshold, the average alpha diversity indices were OTU 29.8, Chao 167.26, ACE 177.46, Shannon 2.79, and Simpson 0.13 in the EG (Table 1). The corresponding indices were OTU 495.33, Chao 673.84, ACE 746.57, Shannon 2.56, and Simpson 0.30 in the CG (Table 2).

**Table 1:** OTUs number and Alpha diversity indices of bacteria in EG group

Sample ID	Sequences	97%					
		Sobs	Shannon	Simpson	Ace	Chao	Coverage
EG1	60451	105	2.585764	0.138376	136.609528	165	0.999079
EG3	56086	111	3.265709	0.077762	125.722103	120.1	0.999484
EG4	58567	133	2.443289	0.207008	197.800314	175	0.998674
EG5	45520	120	3.127874	0.074306	203.872327	201.25	0.999042
EG7	60019	115	2.885793	0.095268	235.351642	214.17	0.998711
EG8	60482	114	2.321447	0.200697	196.435398	163.60	0.998821
EG9	56705	119	2.987039	0.097132	132.647329	134.11	0.999374
EG10	61226	106	2.672341	0.112595	128.060573	129.75	0.999263
EG12	59540	139	2.865805	0.126023	212.80452	187	0.998784
EG14	59297	131	2.792781	0.123471	205.31817	182.67	0.998858

**Table 2:** OTU number and Alpha diversity indices of bacteria in CG group

Sample ID	Sequences	97%					
		Sobs	Shannon	Simpson	Ace	Chao	Coverage
CG2	34741	386	1.083969	0.518271	824.629606	608.04	0.993259
CG6	39502	1333	5.429758	0.024155	1398.130717	1396.49	0.994475
CG11	30885	16	1.170196	0.370826	16.962749	17	0.999926

### Bacterial community composition

#### Phylum level

**Fig. 3:**
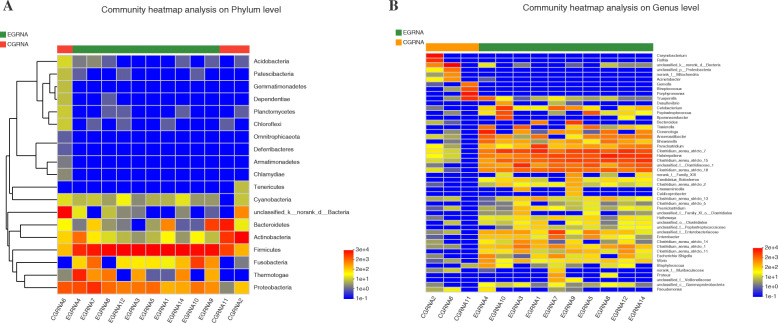
Community heatmap of the phylum and genus level findings in the EG and CG. **A** *Firmicutes*, *Proteobacteria,* and *Thermotogae* were the dominant phyla in the EG. **B** Clostridium _ sensu_ stricto_ 7, *Haloimpatiens*, and *Clostridium*_ sensu_ stricto_ 15 were the dominant genera in the EG. *CG* control group; *EG*, experimental group

At the phylum level, the top six bacteria in the EG were as follows: *Firmicutes* (79.21%), *Proteobacteria* (8.24%), *Thermotogae* (5.74%), *Fusobacteria* (3.43%), *Bacteroidetes* (2.53%), and *Actinobacteria* (0.78%), which accounted for 99.93% of the total population of bacteria (Table 3). In the CG, the top five phyla were *Actinobacteria* (41.07%), *Bacteroidetes* (17.44%), *Firmicutes* (7.3%), *Proteobacteria* (4.4%), and *Acidobacteria* (0.04%) (Table 4). Similar trends were noted in the community heatmap (Fig. 3A).

**Table 3:** Bacterial community composition of phylum level in the EG group

Phylum level	EG1	EG3	EG4	EG5	EG7	EG8	EG9	EG10	EG12	EG14	Average
Firmicutes	94.76%	92.08%	35.06%	92.39%	57.38%	86.97%	72.09%	72.44%	94.69%	94.22%	79.21%
Proteobacteria	1.23%	5.64%	14.64%	7.21%	31.64%	7.39%	2.86%	5.56%	4.73%	1.51%	8.24%
Thermotogae	0.00%	1.95%	42.77%	0.00%	2.64%	5.47%	1.36%	0.00%	0.00%	3.19%	5.74%
Fusobacteria	0.14%	0.29%	0.53%	0.33%	7.37%	0.00%	2.59%	21.76%	0.38%	0.94%	3.43%
Bacteroidetes	3.61%	0.00%	0.01%	0.02%	0.61%	0.01%	21.04%	0.00%	0.01%	0.00%	2.53%
Actinobacteria	0.22%	0.01%	6.83%	0.03%	0.31%	0.08%	0.04%	0.22%	0.03%	0.01%	0.78%

**Table 4:** bacterial community composition of phylum level in the CG group

Phylum level	CG2	CG6	CG11	Average
Actinobacteria	94.41%	0.56%	28.24%	41.07%
Unclassified	3.43%	85.44%	0.00%	29.62%
Bacteroidetes	0.12%	0.22%	51.97%	17.44%
Firmicutes	1.18%	0.95%	19.78%	7.30%
Proteobacteria	0.77%	12.43%	0.01%	4.40%
Acidobacteria	0.04%	0.08%	0.00%	0.04%

#### Genus level

At the genus level, the top six bacteria in the EG were as follows: *Clostridium*_sensu_stricto_7 (16.71%), *Haloimpatiens* (15.09%), *Clostridium*_sensu_stricto_15 (12.83%), *Clostridiaceae*_1 (8.21%), *Clostridium*_sensu_stricto_18 (6.58%), and *Oceanotoga* (5.74%), which accounted for 65.16% of the bacterial population (Table 5). The top five genera were *Corynebacterium* (29.62%), *Porpphyromonas* (22.11%), *Trueperella* (9.42%), *Rothia* (9.34%), and *Streptococcus* (4.90%) in the CG (Table 6). The trend was consistent with that noted in the community heatmap at the genus level (Fig. 3B).

**Table 5:** bacterial community composition of genus level in EG group.

Genus level	EG1	EG3	EG4	EG5	EG7	EG8	EG9	EG10	EG12	EG14	average
Clostridium_sensu_stricto_7	33.06%	14.90%	4.72%	20.45%	5.18%	8.12%	17.13%	23.53%	26.67%	13.32%	16.71%
Haloimpatiens	11.15%	4.72%	3.97%	12.09%	16.32%	39.60%	12.82%	2.45%	25.30%	22.52%	15.09%
Clostridium_sensu_stricto_15	7.02%	6.34%	1.77%	14.42%	12.13%	18.88%	17.77%	8.68%	14.53%	26.74%	12.83%
Clostridiaceae_1	2.12%	32.25%	2.17%	12.49%	6.76%	1.55%	5.04%	1.53%	7.32%	10.88%	8.21%
Clostridium_sensu_stricto_18	6.69%	7.47%	2.91%	5.53%	10.26%	9.09%	6.76%	3.85%	4.15%	9.12%	6.58%
Oceanotoga	0.00%	1.95%	42.77%	0.00%	2.64%	5.47%	1.36%	0.00%	0.00%	3.19%	5.74%

**Table 6:** Bacterial community composition of genus level in the CG group

Genus level	CG2	CG6	CG11	Average
Unclassified	3.43%	85.44%	0.00%	29.62%
Corynebacterium	66.32%	0.00%	0.00%	22.11%
Porphyromonas	0.00%	0.00%	51.97%	17.32%
Trueperella	0.00%	0.01%	28.24%	9.42%
Rothia	28.01%	0.00%	0.00%	9.34%
Streptococcus	0.00%	0.02%	14.69%	4.90%

### Community composition difference analysis

**Fig. 4:**
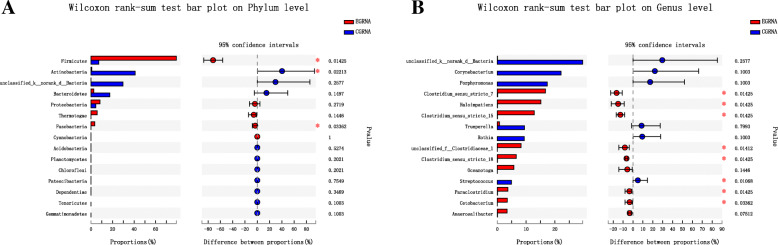
Wilcoxon rank-sum test for phylum and genus level findings between the EG and CG. At the phylum level, *Firmicutes* was the most abundant in the EG (*P* = 0.014), while *Actinobacteria* showed the highest prevalence in the CG (*P* = 0.022). At the genus level, *Clostridium*_sensu_stricto_7, *Haloimpatiens*, and *Clostridium*_sensu_stricto_15 were the most abundantly detected in the EG (all *P* < 0.05). *EG*, experimental group; *CG*, control group

Using the Wilcoxon rank-sum test, significant differences in the bacterial community composition were observed between the two groups at the phylum and genus levels. At the phylum level, *Firmicutes* was obviously the most abundantly observed type in the EG (*P* = 0.014), while *Actinobacteria* were more frequently noted in the CG (*P* = 0.022). At the genus level, *Clostridium*_sensu_stricto_7, *Haloimpatiens*, and *Clostridium*_sensu_stricto_15 were more commonly seen in the EG (all *P* < 0.05) (Fig. 4).

### Culture and identification of bacteria

**Fig. 5:**
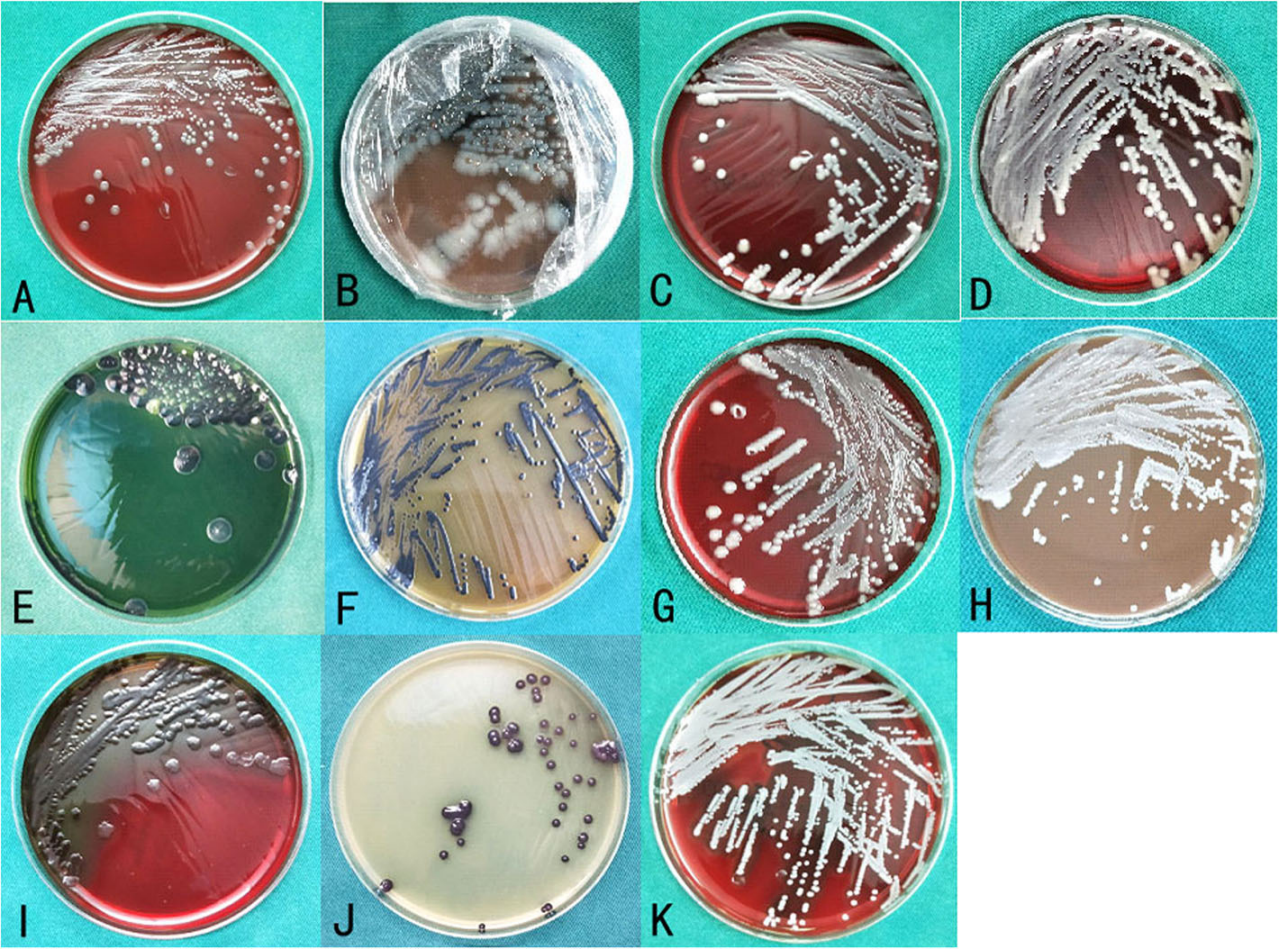
Bacterial culture in the EG. **A**–**K** present the types of bacteria in the EG, corresponding to *Vibrio vulnificus*, *Vibrio cholerae* (non-O1, non-O139) (sealed and photographed due to the infectivity and risk of *Vibrio cholerae*), *Enterobacter cloacae, Klebsiella pneumoniae*, *Proteus mirabilis*, *Citrobacter brucella*, *Escherichia coli*, *Enterococcus faecalis*, *Pseudomonas aeruginosa*, *Shewanella alginate*, and *Staphylococcus aureus. EG*, experimental group

A total of 45 bacterial strains were isolated in the EG; separate results are presented in Fig. 5. In summary, *S. algae*, *S. aureus*, and *K. pneumoniae* showed the greatest predominance, accounting for 17.78% of the total population. The second most commonly observed pathogen was *E. coli* (13.34%). Four strains of *Enterobacter cloacae* were isolated, accounting for of the 8.89% bacteria. *V. vulnificus* accounted for 6.67% of the population (Table 7).

**Table 7:** Comparison of bacterial culture between SG group and EG group

Bacteria family/genus	Species	SG group (*N* = 53)	EG group (*N* = 45)	*P* value
Vibrio	Vibrio alginolyticus	8(15.10)	0(0)	0.007
	Vibrio parahaemolyticus	6(11.32)	0(0)	0.03
	Vibrio fluvialis	6(11.32)	0 (0)	0.03
	Vibrio vulnificus	0(0)	3(6.67)	> 0.05
	Vibrio cholerae (not O1 and not O139)	0(0)	2(4.44)	> 0.05
Enterobacteriaceae	Enterobacter cloacae	7(13.22)	4(8.89)	> 0.05
	Klebsiella pneumoniae	5(9.43)	8(17.78)	> 0.05
	Proteus vulgaris	5(9.43)	0(0)	> 0.05
	Photobacterium damselae	5(9.43)	0(0)	> 0.05
	Proteus mirabilis	0(0)	2(4.44)	> 0.05
	Citrobacter braakii	0(0)	1(2.22)	> 0.05
	Escherichia coli	0(0)	6(13.34)	0.008
Non fermenting bacteria	Pseudomonas aeruginosa	6(11.32)	2(4.44)	> 0.05
	Shewanella algae	5(9.43)	8(17.78)	> 0.05
Gram positive cocci	Staphylococcus aureus	0(0)	8(17.78)	0.001
	Enterococcus faecalis	0(0)	1(2.22)	> 0 .05

**Fig. 6:**
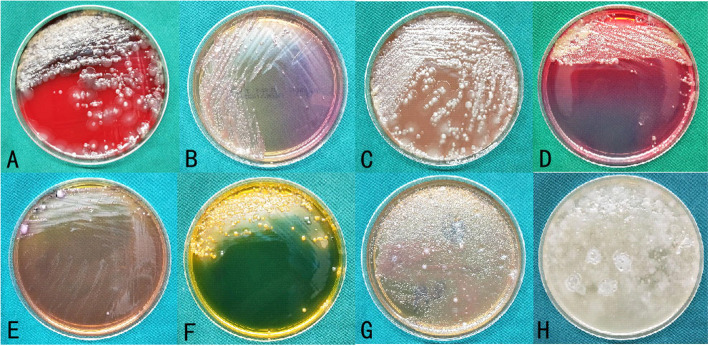
Bacterial culture in the SG. **A**–**H** present the following information: blood plate, MacConkey plate, chocolate plate, Chinese blue agar plate, SS plate, TCBS agar plate, Baird-Parker plate, and 3% sodium chloride tryptone soy agar plate. *TCBS*, thiosulfate citrate bile salts sucrose; *SG*, seawater group; *SS*, Salmonella–Shigella

A total of 53 bacterial strains were isolated in the SG, with *Vibrio alginolyticus* showing the strongest predominance, accounting for 15.10% of the population, followed by *E. cloacae* (13.22%). The detection rates of *Vibrio parahaemolyticus*, *Vibrio fluvialis*, and *P. aeruginosa* were 11.32% each (Table 7 and Fig. 6).

The EG showed a higher detection rate of *E. coli* and *S. aureus* than the SG (*P* = 0.008 and *P* = 0.001, respectively). However, the detection rates of *V. alginolyticus*, *V. parahaemolyticus*, and *V. fluvialis* were higher in the SG than the EG (*P* = 0.007, *P* = 0.03, and *P* = 0.03, respectively) (Table 7).

## Discussion

In our study, we found that the infection rate was 100%, and mortality value was 20% following ocean bacterial infection. Both light microscopy and SEM showed that a large number of bacteria were distributed in the medullary cavity after ocean bacterial infection. Illumina MiSeq sequencing demonstrated that in the EG, *Firmicutes, Proteobacteria, Thermotogae, Fusobacteria, Bacteroidetes*, and *Actinobacteria* were the dominant bacteria at the phylum level. At the genus level, *Clostridium*_sensu_stricto_7, *Haloimpatiens, Clostridium*_sensu_stricto_15, *Clostridiaceae*_1, *Clostridium*_sensu_stricto_18, and *Oceanotoga* were the most commonly noted. Following the bacterial culture of the obtained medullary cavity specimens, *K. pneumoniae*, *S. algae*, *S. aureus*, *E. coli*, *E. cloacae*, and *V. vulnificus* were identified as the main infective species. Moreover, we also found that the detection rates of *V. alginolyticus*, *V. parahaemolyticus*, and *V. fluvialis* were not high in the EG, inconsistent with the findings in the SG.

There are about 100 million bacteria present in every liter of seawater, at all depths and latitudes. Exposure to bacteria-rich solutions greatly promotes the entry of pathogens, and the infection rate associated with wounds exposed to seawater seems to be higher than that related to ground wounds [21]. Different ocean bacterial illness incidence and death rates are reported every year, globally. In the USA, annually, an estimated 8000 people experience an illness caused by *Vibrio*, which is the predominant genus in the ocean [22]. Zhu et al. [7] reported that 8% of all open fracture infections are associated with exposure to seawater. In a survey conducted by Hlady and Klontz [23], 6.5% of wound infections were found to be caused by *V. cholerae* non-O1 strains, resulting in a case fatality rate of 5%. Levine et al. [24] reported that of the 121 patients with an ocean bacterial infection, 19 were hospitalized, and two died. These events usually occur during warm-water months, between April and October [23]. A majority of these infections were observed in patients with wounds that were exposed to seawater within 7 days. In this experiment, 1 week after operation, the infection and mortality values in the EG were 100% and 20%, indicating that wounds infected by seawater bacteria are associated with very high infection rates in the absence of treatment. We also observed local infection, which leads to the deterioration of systemic conditions and results in death.

*Clostridium*, which belongs to the phylum *Firmicutes*, was the dominant bacteria in the infective medullary cavity, based on the Illumina MiSeq sequencing performed in our study. *Clostridium*, as a genus, comprises 130 species of bacteria, including a large group of anaerobic and slightly aerobic coarse bacilli, of which *C. tetanus*, *C. perfringens*, *C. botulinum*, and *C. difficile* have been extensively investigated. The distribution of *Clostridium* is weaker than that of *Vibrio* in the sea, with a majority of *Clostridium* spp. reported as existing as parasites on shellfish and zooplankton [25–28]. A majority of the pathogenic bacteria in seawater belong to the *Vibrio* spp. and *Aeromonas* spp., in addition to *Erysipelothrix rhusiopathiae*, *Mycobacterium marinum*, and *Streptococcus iniae*, among others; no reports of *Clostridium* infection from seawater have been published to date [29, 30]. In our study, although *Clostridium* accounted for less than 0.5% of the bacteria detected in seawater, the closed tibial medullary cavity in our animal models created an anaerobic environment, and none of the experimental animals were treated with antibiotics. However, *Clostridium* can grow rapidly to become the dominant pathogen in the anoxic environment of the tibial medullary cavity, causing local tissue necrosis and death after systemic infection. In our study, EG3 died of infection. In this case, the wound smelled of decay and when the incision was opened, we observed that the soft tissue was black in color and odorous, in line with the clinical symptoms of anaerobe infection. Therefore, although few studies have focused on the anaerobic infection caused by wound contact with seawater, for deep wounds, it is still necessary to perform complete debridement and provide a sufficient amount of antibiotics for the prevention of anaerobe infection.

In the present study, *S. algae, S. aureus*, *K. pneumoniae*, *E. coli*, and *Enterobacter cloacae* were the most predominant pathogens in bacteria culture of the EG, accounting for approximately 80%.

*S. algae* is a rod-shaped Gram-negative marine bacterium often found in warm climates in a marine environment [31]. However, *S. algae* is a rare human pathogen, and symptoms of *S. algae* infection are often misidentified as other bacteria [32]. However, reports of *S. algae* infection have been increasing in recent years, including chronic skin ulcer [33], necrotizing fasciitis [34, 35], chronic liver disease [36], spondylodiscitis [37], osteomyelitis [38], and others. *S. algae* infections often occurred via ulcers or wounds in a marine environment [39, 40]. With the increasing chance of contact with seawater, including the development of ocean tourism and beach entertainment, the chance of marine bacterium infection will gradually increase. Moreover, with global trends of warmer water temperatures, *S. algae* infection may continue to increase in the future, especially in tropical areas such as India and China.

*S. aureus* is a Gram-positive, round-shaped bacterium frequently found in the upper respiratory tract, skin, and gut mucosa. *K. pneumoniae*, *E. coli*, and *Enterobacter cloacae* belong to the Enterobacteriaceae family and can be found in human intestines. With increasing human activities on coastal beaches, the number of intestinal bacteria in the coastal seawater has gradually increased. It has been reported that in Xiamen, China, seven pathogenic marker genes were found in beach-bathing waters at 13 locations; this prior study targeted intestinal bacteria and human fecal markers, which were found in almost all samples [41]. Similar contamination was also found in Rouge River in Canada [42] and Florida Gulf coast beaches [43]. Deterioration and pollution of coastal water environment are directly related to the occurrence of these pathogens and their infectious diseases. Pathogen from water environments often through the fecal–oral route or wound-causing infections [44]. Our samples were also collected from coastal areas where human activities are frequent; it is inevitably experiencing intestinal bacteria contamination. In addition, the enclosed space of the bone marrow cavity also creates a suitable environment for rapid growth of facultative anaerobic bacteria.

The detection rates of *V. alginolyticus*, *V. parahaemolyticus,* and *V. fluvialis* were higher in the SG than in the EG (all *P* < 0.05, respectively) by bacterial culture. *V.* alginolyticus accounts for the largest number of Gram-negative bacteria in the Vibrio family and is widely distributed in various sea and coastal areas. It is a conditional pathogen in marine animals, which can cause otitis media, gastroenteritis, food poisoning, and septicemia in the human body. However, cases of *V. alginolyticus* bone infection have been rarely reported [45, 46]. In our study, the body temperature of the rabbits was 38.1–39.2 °C; *V. alginolyticus* survives at temperatures of 17–35°C [47]. This may explain why this bacterium was not observed in the tibial medullary cavity in the EG.

*V. parahaemolyticus* is a commonly reported worldwide cause of food poisoning and leads to diarrhea and acute gastroenteritis [48]. This organism is associated with an annual infection number of 99,351 [49]. The ingestion of raw or undercooked seafood is a major risk factor for infection. The contamination rates of *V. parahaemolyticus* are 19.33% and 10.67% in live shrimp and fish, respectively [50].

*V. fluvialis* is a halophilic Gram-negative bacterium, which has a curved cell morphology and polar flagella. In many investigations, the detection frequency of *V. fluvialis* was very high in marine mollusks, predominantly in bivalves [51, 52]. Similar to *V. parahaemolyticus*, *V. fluvialis* predominantly causes gastroenteritis and acute diarrhea [53, 54].

The reasons for the differences in the detection rates of *V. parahaemolyticus* and *V. parahaemolyticus* between the SG and EG may be that the enrichment of bacteria in seawater and seawater filter membranes is not absolutely average. *V. parahaemolyticus* and *V. parahaemolyticus* were found in the SG but not in the EG. Moreover, *V. parahaemolyticus* and *V. fluvialis* are halophilic, and the medullary cavity of rabbit tibia is not suitable for growth.

This study has some limitations. First, the seawater sampling was performed at a single location, and the samples could not have covered all kinds of ocean bacteria. However, in further investigation, our research will expand to other coastal area to cover more ocean bacteria. Second, the number of experimental animals was insufficient, although it met the requirements of biological repeatability (n ≥ 3). In future research, we will increase the number of experimental animals. Finally, Illumina MiSeq sequencing could only provide detection results at the genus level. However, bacterial culture could remedy this defect and allow for the observation of the pathogenic bacterial species.

## Conclusion

The present study is the first to combine SEM, Illumina MiSeq sequencing, and bacterial culture to explore the detection rates of ocean bacterial infections using a rabbit model. SEM showed that a large number of bacteria were distributed in the medullary cavity after ocean bacterial infection. Illumina MiSeq sequencing found that *Firmicutes* and *Clostridium* were the dominant bacteria in the experimentally treated rabbits. Following bacterial culture of the medullary cavity specimens, *K. pneumoniae*, *S. algae, S. aureus*, *E. coli*, *E. cloacae,* and *V. vulnificus* were identified as the main infective species. In summary, the model we constructed was comprehensively evaluated using four techniques: histopathology and SEM, gene detection, and plate culture, which provided a scientific basis for the clinical diagnosis and treatment of patients in such settings.

## Supplementary Information

**Additional file 1.**

**Additional file 2.**

**Additional file 3.**

**Additional file 4.**

**Additional file 5.**

**Additional file 6.**

**Additional file 7.**

**Additional file 8.**

**Additional file 9.**

**Additional file 10.**

**Additional file 11.**

**Additional file 12.**

**Additional file 13.**

## Data Availability

The datasets used and analyzed during the current study are available from the corresponding author on reasonable request.

## Abbreviations

SG: Seawater group
EG: Experience group
CG: Control group
SEM: Scanning electron microscopy
HE: Hematoxylin & eosin
PCR: Polymerase chain reaction
OUT: Operational taxonomic unit
TCBS: Thiosulfate citrate bile salts sucrose
SS: Salmonella–Shigella
*K.*: *Klebsiella*
*S. algae*: *Shewanella alga*
*S. aureus*: *Staphylococcus aureus*
*E.*: *Escherichia*
*C.*: *Clostridium*
*V.*: *Vibrio*
spp.: Species
